# The complete mitochondrial genome of a marine triclad, *Paucumara falcata* (Platyhelminthes, Tricladida, Maricola)

**DOI:** 10.1080/23802359.2026.2652593

**Published:** 2026-04-07

**Authors:** Liyuan Chen, Yuanyuan Liao, Weixuan Li, Junyu Li, Antai Wang, Yu Zhang

**Affiliations:** a Shenzhen Key Laboratory of Marine Bioresource and Eco-environmental Science, College of Life Sciences and Oceanography, Shenzhen University, Shenzhen, Guangdong, PR China; b Division of Life Science, The Hong Kong University of Science and Technology, Hong Kong SAR, China; c State Key Laboratory of Protein and Plant Gene Research, Center for Bioinformatics, School of Life Sciences, Peking University, Beijing, PR China; d Department of Ocean Science, Hong Kong University of Science and Technology; e Guangdong Engineering Research Center for Marine Algal Biotechnology, College of Life Sciences and Oceanography, Shenzhen University, Shenzhen, Guangdong, PR China

**Keywords:** Mitogenome, molecular phylogeny, marine planarian, gene order

## Abstract

The marine triclad *Paucumara falcata* Wang & Li, 2019 exhibitslow-salinity tolerance, making it a valuable model for understanding the evolution of freshwater adaptation in Tricladida. Here we assembled its complete circular mitogenome (13,247 bp), which contains two rRNAs, 12 PCGs, and 22 tRNAs. Phylogenetically, *P. falcata* forms a well-supported clade with two other Maricolan species. Comparative analysis revealed that the three Maricolan species sequenced to date each exhibit a unique arrangement, while mitochondrial gene order is highly conserved within Continenticola. These findings highlight the need for additional mitogenomic data to resolve triclad phylogeny and elucidate the evolution of gene order in Maricola.

## Introduction

*Paucumara falcata* Wang & Li, 2019, a marine planarian classified into the genus *Paucumara* (Platyhelminthes, Tricladida, Maricola), is well adapted to low salinity and even freshwater (Chen et al. 2019). The salinity tolerance is similar not only to its congener *P. trigonocephala* and *P. mentulalacertosa* (Sluys 1989; Li et al. 2021), but also to distant species, such as *Miroplana shenzhensis* (Yu et al. 2013; Huang et al. 2022), *Pentacoelum kazukolinda* and *Pentacoelum hispaniense* (Sluys et al. 2015). It remains unclear whether freshwater adaptation in Tricladida is a single or multiple evolutionary events, and thus it is important for future studies to elucidate the evolutionary relationship of these species in Tricladida.

However, previous phylogenetic analyses were mainly conducted on single or a few genes, which might not provide sufficient information for this purpose (Stocchino et al. 2021). In this study, we report the complete mitogenome of *P. falcata*, providing the third genomic resource for Maricola to support future phylogenetic and evolutionary investigations.

## Materials and methods

**Figure 1.**
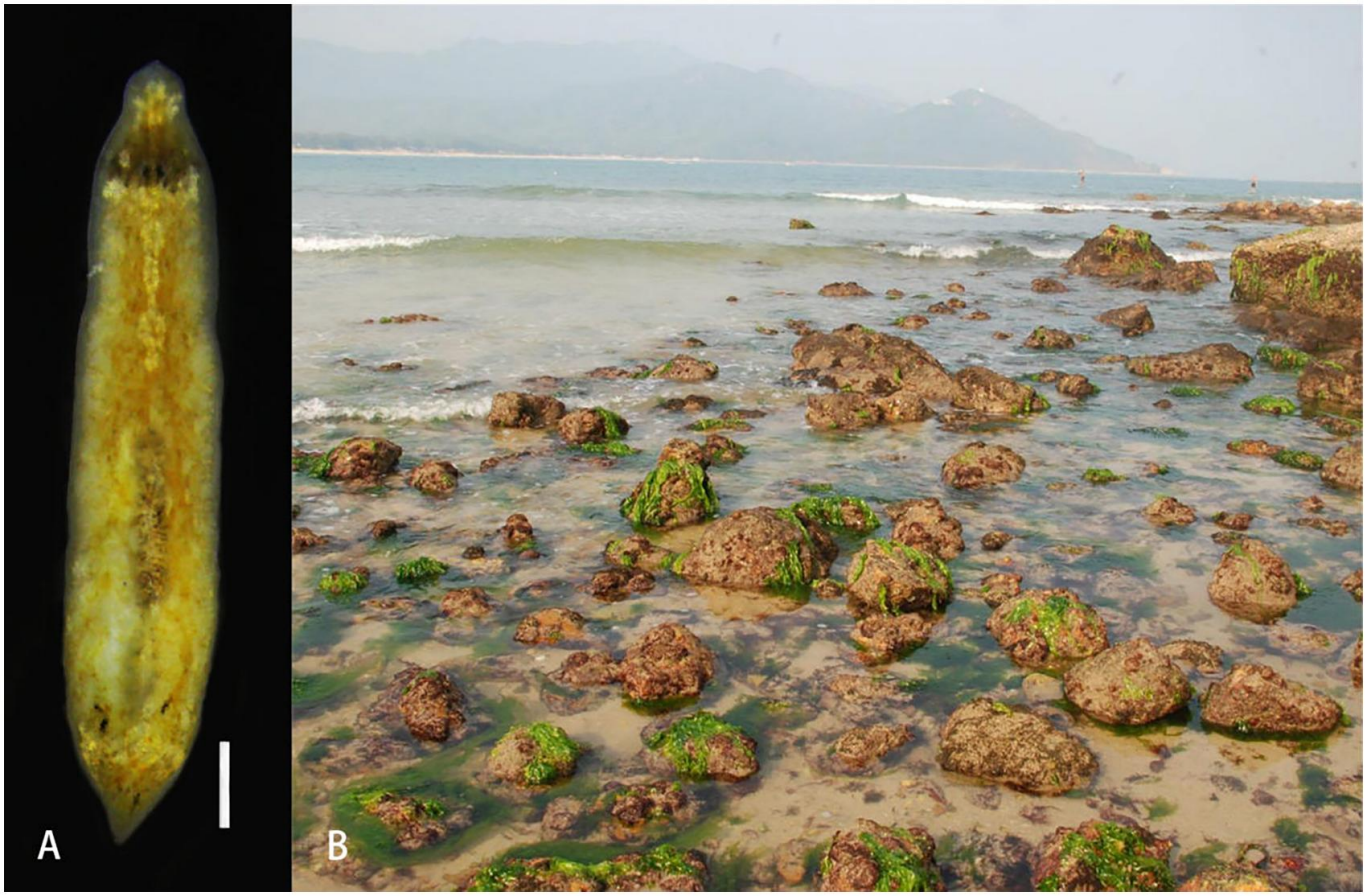
Specimen and habitat of *P. falcata*, photographed by Liyuan Chen. A) Dorsal view of the living specimen (scale bar = 1 mm). External morphological features: triangular head; three pale yellow transverse pigmentation bands, at the anterior body margin, immediately behind the eyes and across the tail end of the body; a brown band extends anteriorly from eyes (Chen et al. 2019). B) the collection site, a rocky intertidal zone at Dapeng Bay, Shenzhen, China.

The *P. falcata* specimen was collected from a beach in eastern Shenzhen, Guangdong, China (22°28′N, 114°31′E) on 29 April 2017 by Chen et al. (2019) and subsequently reared in the laboratory. The living image of this sample is provided in Figure 1. The genomic DNA was extracted from the anterior body region of the specimen by E.Z.N.A.^TM^ Mollusk DNA Isolation Kit (Omega, Norcross, GA, USA). The remaining specimen was preserved in 95% ethanol and deposited as a voucher at the College of Life Sciences and Oceanography, Shenzhen University (voucher no. Pf001). Species identification was based on external morphology and 18S rDNA sequencing. REPLI-g Midi Kit (QIAGEN, Hilden, Germany) was used to amplify the genomic DNA and conducted paired-end sequencing on the Illumina Hiseq 2500 platform (Novogene, Beijing, China). The mitogenome sequences were assembled and annotated using MitoFlex v.0.2.9 (Li et al. 2021) and the annotation result was confirmed by MITOS (Bernt et al. 2013).

The phylogeny was inferred using a dataset concatenates 12 PCGs from 15 Tricladida species, while two species of Polycladida were included as outgroup (Figure 3). Multiple sequence alignments (MSA) were performed by MACSE v2.03 (Ranwez et al. 2018). The processed sequences were subsequently trimmed using Gblocks v0.91b (Talavera and Castresana 2007). The substitution saturation test for the first and second positions of all protein coding genes (PCGs) is carried out by DAMBE6 software (Xia et al. 2003; Xia and Lemey 2009). PartitionFinder2 (Lanfear et al. 2017) was used to determine the best-fit partitioning scheme and substitution models. The phylogenetic trees were constructed by IQ-TREE v2.1.2 (Chernomor et al. 2016; Minh et al. 2020) performing standard bootstrap analysis with 10,000 replications for Maximum Likelihood (ML), and Mrbayes v. 3.2.6 (Ronquist et al. 2012) applied with 5,000,000 generations, sampling every 5,000 generations for Bayesian Inference (BI).

## Results

**Figure 2.**
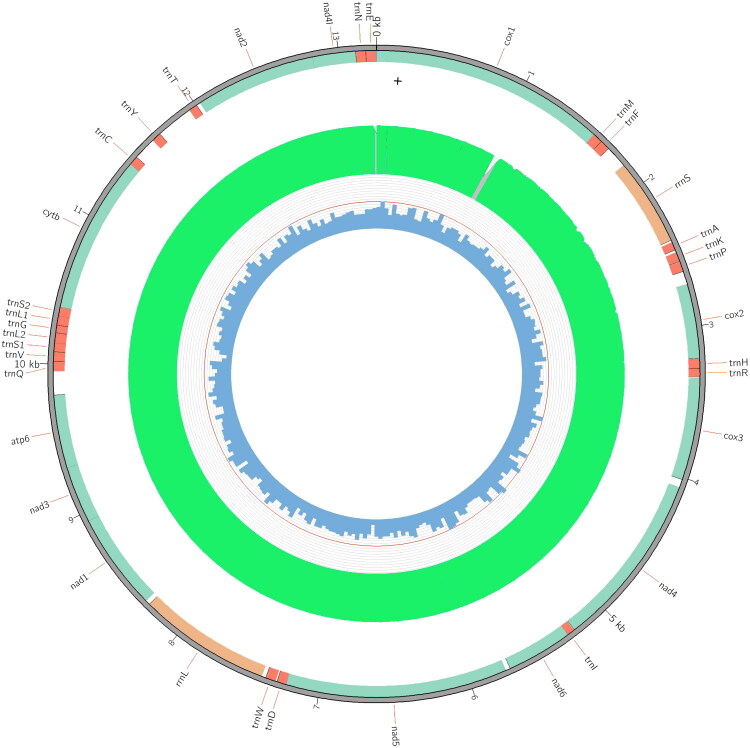
The mitochondrial genome of *Paucumara falcata*. Outer circle: annotation of genes, with protein-coding genes, ribosomal RNAs and transfer RNAs represented by cyan, orange and red, respectively. Intermediate circle: Depth of coverage, green color represents coverage is greater than 95% of average coverage. Inner circle: GC content, while the orange circle indicates 50%.

The assembled, circular mitogenome of *P. falcata* (GenBank accession number: OM371324, Figure 2) is 13,247 bp in length, and codes for 12 PCGs, two rRNAs and 22 tRNAs. According to the automatic annotation by MITOS (Bernt et al. 2013) and MitoFlex (Li et al. 2021), *ATP8* is absent in the mitogenome of *P. falcata*, a condition previously reported in other flatworm mitogenomes (e.g. *Obrimoposthia wandeli*, Yang et al. 2019; *Miroplana shenzhensis*, Huang et al. 2022). All mitochondrial genes were transcribed from the same strand. The PCG order of *P. falcata* is *COX1-COX2-COX3-ND4-ND6-ND5-ND1-ND3-ATP6-CYTB-ND2-ND4L*. The nucleotide base composition is 29.4% A, 12.9% C, 20.4% G, and 37.3% T, with a total A + T content of 66.7%. The uniformity of sequencing coverage across the mitogenome was confirmed by depth analysis (Supplementary Figure S1).

**Figure 3.**
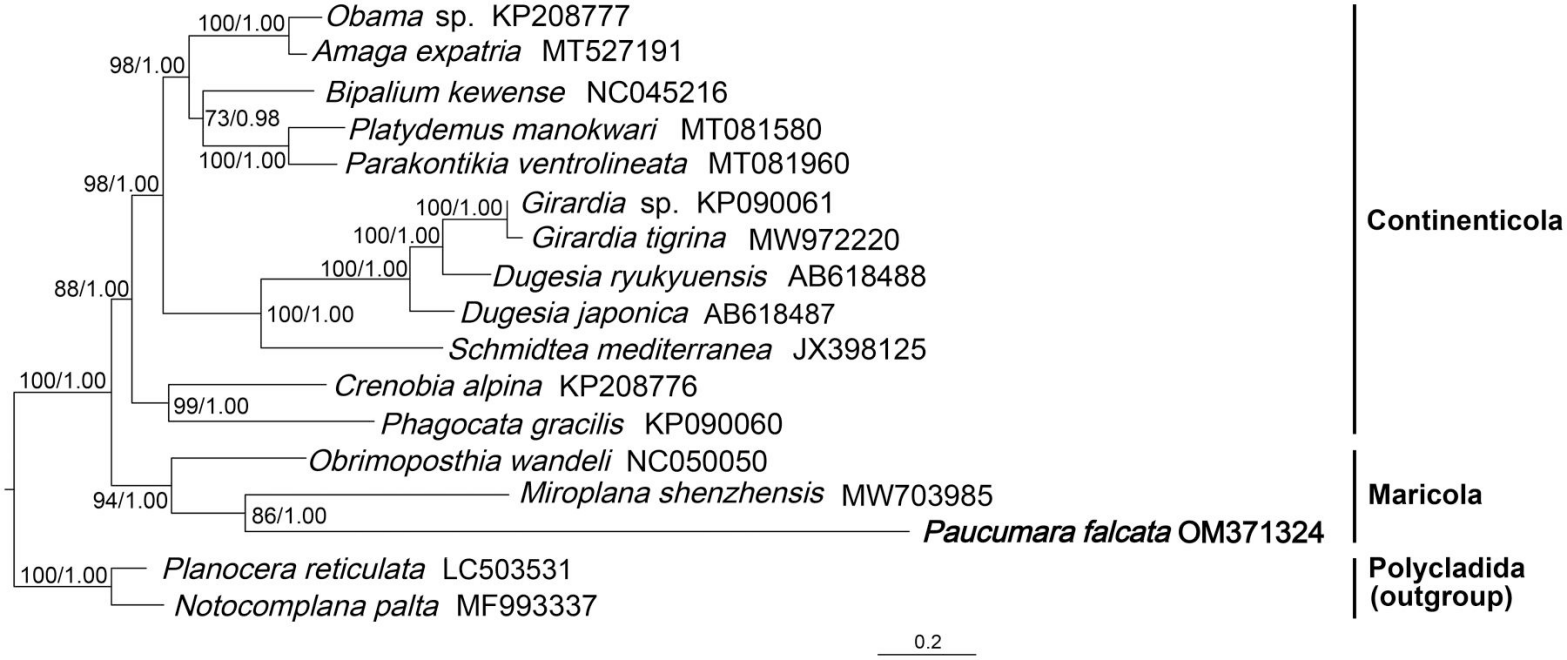
Maximum likelihood phylogenetic tree topology inferred from 12 PCGs. Numbers at nodes indicate support values (bootstrap/posterior probability). The following sequences were used: *Obama* sp. (KP208777; Solà et al. 2015), *Amaga expatria* (MT527191; Justine et al. 2020), *Bipalium kewense* (NC_045216; Gastineau et al. 2019), *Platydemus manokwari* (MT081580; Gastineau et al. 2020), *Parakontikia ventrolineata* (MT081960; Gastineau and Justine 2020), *Girardia* sp. (KP090061; Ross et al. 2016), *Girardia tigrina* (MW972220; Cheng et al. 2021), *Dugesia ryukyuensis* (AB618488; Sakai and Sakaizumi 2012), *Dugesia japonica* (AB618487; Sakai and Sakaizumi 2012), *Schmidtea mediterranea* (JX398125; Ross et al. 2016), *Crenobia alpina* (KP208776; Solà et al. 2015), *Phagocata gracilis* (KP090060; Ross et al. 2016), *Obrimoposthia wandeli* (NC_050050; Yang et al. 2019), *Miroplana shenzhensis* (MW703985; Huang et al. 2022), *Paucumara falcata* (OM371324; this study), *Planocera reticulata* (LC503531; Yonezawa et al. 2020), and *Notocomplana palta* (MF993337; Kenny et al. 2019).

Phylogenetic trees inferred by both methods exhibited the same topology (Figure 3), in which *P. falcata* constituted a well-supported clade with *M. shenzhensis* and *Obrimoposthia wandeli* belonging to the suborder Maricola. However, *P. falcata* exhibited a long branch on the tree.

## Discussion

*Paucumara falcata* shares a sister-group relationship with another freshwater-adapted species *M. shenzhensis*, which differs from the results inferred from ribosomal DNA (Li et al. 2019: Figures 1 and 2), in which it shares closer relationship with *O. wandeli.* The long branch observed for *P. falcata* may indicate an elevated evolutionary rate in this lineage, or could be influenced by limited taxon sampling within Maricola, which might raise the possibility of long-branch attraction artifacts (Bergsten 2005). In the future, more mitogenome sampling in Maricola will be required to confirm its branch length and actual evolutionary rate.

The mitochondrial gene order is considered highly conserved in Continenticolans (Ross et al. 2016), with identical arrangement not only for PCGs (*COX1, ND6-ND5, COX2, ND3, ND2, ND4L-ND4*) but also for some tRNA genes (*S2-D-R*, *I-Q-K*, *V*) (Supplementary Figure S2). In *P. falcata*, except for the *ND6-ND5* gene block, the PCG arrangement differs from those of Continenticolans (Supplementary Figure S2). However, the *ND6-ND5* gene block is not a highly conserved character in Maricola, as in *M. shenzhensis*, *ND6* and *ND5* are separated by *ND3* and *ND2* (Supplementary Figure S2). In addition, diverse tRNA rearrangements have been found among the three Maricolans (Supplementary Figure S2), while the tRNA arrangements of Maricolans also differ from those of Continenticolans (Supplementary Figure S2). Therefore, Maricolans exhibit higher diversity in gene order than Continenticolans.

The various gene orders of Maricolans could be generated either before or after the divergence between Maricolans and Continenticolans. However, it’s still unclear which suborder is more ancestral. As phylogeny based on ribosomal genes shows, Continenticola branches out first among three suborders of Tricladida (Stocchino et al. 2021). In this scenario, the diversification on mitochondrial gene order of Maricolans possibly occurred after their divergence, and could be related to marine adaptation. However, there’s no genomic or mitogenomic data giving further information on the phylogenetic relationship within Tricladida. To have a thorough understanding on the origin and evolution of triclad planarians, more systematic and comprehensive mitogenomic analysis, especially for the less known suborders Maricola and Cavernicola, are required.

## Supplementary Material

Supplementary material.doc

## Data Availability

The genome sequence data that support the findings of this study are openly available in Genbank of NCBI at https://www.ncbi.nih.gov under the accession no. OM371324. The associated BioProject, SRA, and Bio-Sample numbers are PRJNA832536, SRR18935420, and SAMN27865206 respectively.
