# Supplementary material for: The complete mitochondrial genome of a marine triclad, Paucumara falcata (Platyhelminthes, Tricladida, Maricola)
Source: Mitochondrial DNA B Resour. 2026 Apr 7;11(5):594–8. doi: 10.1080/23802359.2026.2652593 (PMC13059034; doi:10.1080/23802359.2026.2652593)

**Supplementary material**

**Figure S1.** Coverage depth across the *Paucumara falcata* mitogenome. The x-axis represents genome position (bp), and the y-axis represents sequencing depth. Sequencing reads were mapped to the assembled mitogenome, yielding a mean depth of 7,926×. Per-base depth was extracted with SAMtools and visualized using R (ggplot2).


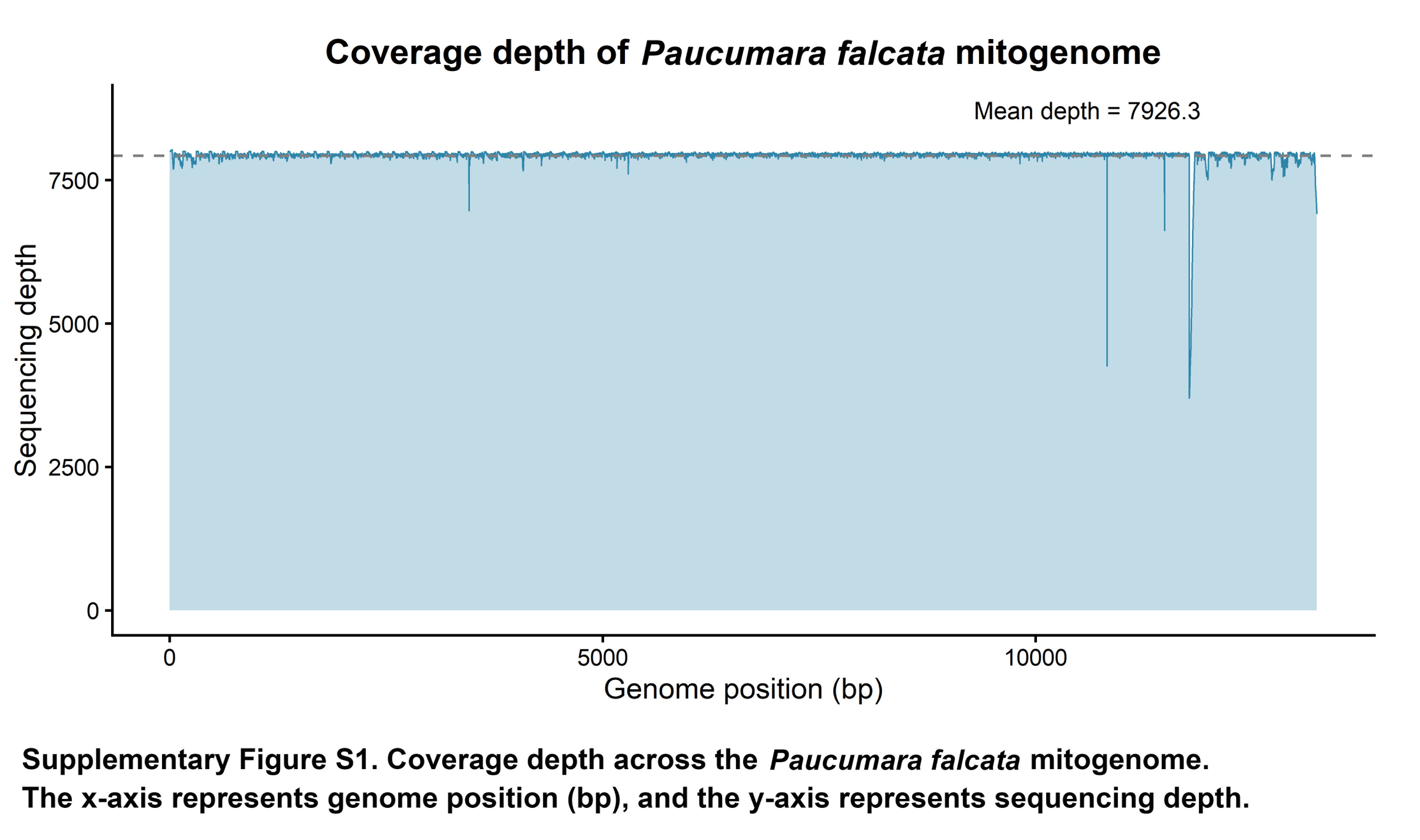


**Figure S2.** Mitochondrial gene order in Tricladida (Maricola and Continenticola)


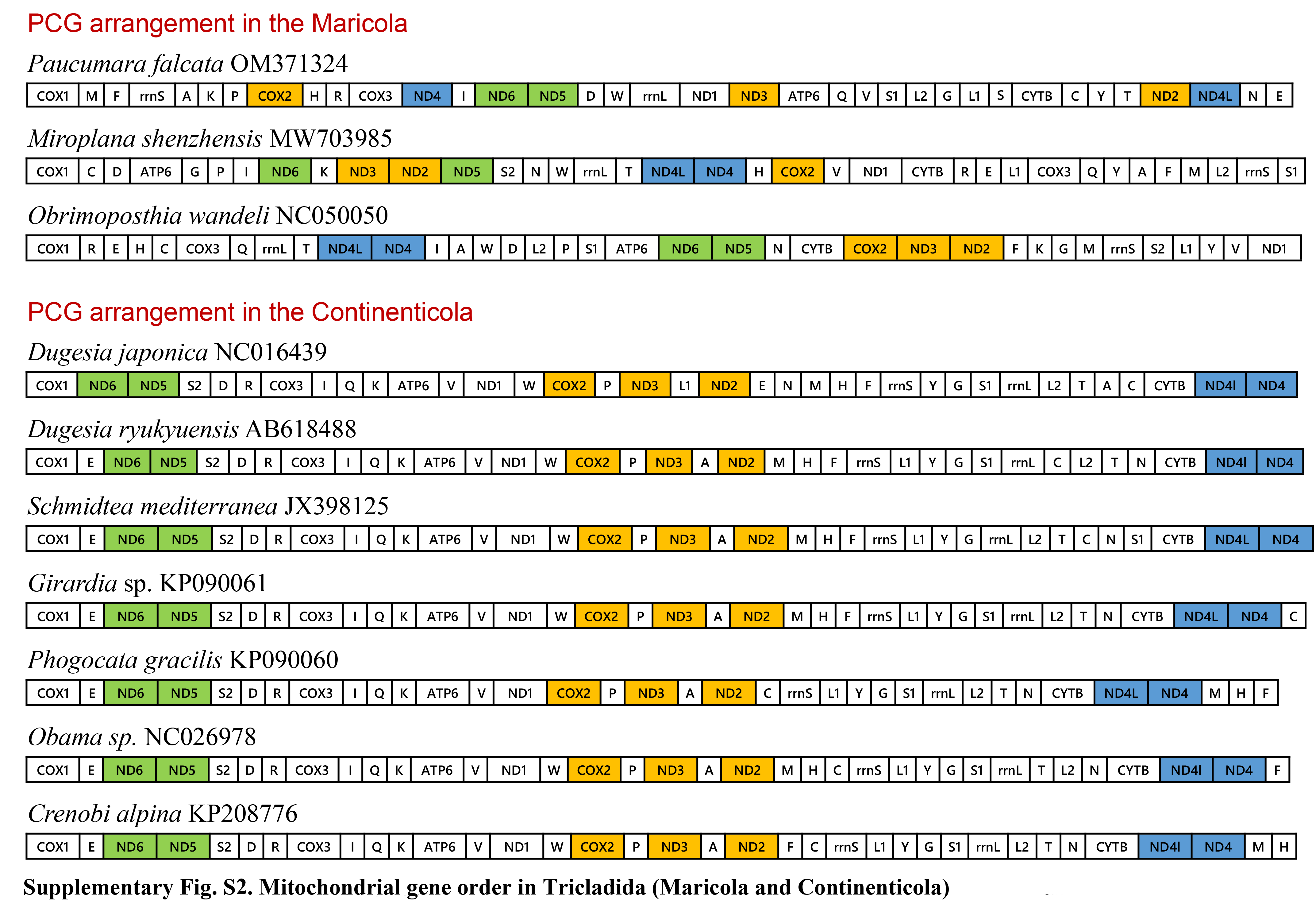

Supplement: Supplementary material.doc [file TMDN_A_2652593_SM0680.doc]
